# Eating by Rule

**Published:** 1858-08

**Authors:** 


### EATING BY RULE.

SCIENTIFIC investigation assures us, that "the amount of nourishment required by an animal for its support must be in a direct ratio with the quantity of oxygen taken into the system;" which, being put into homely English, means, that as our supply of oxygen comes from the air we breathe, it follows, that the more pure air we inhale, the more oxygen we consume; it then follows, necessarily, as out-door air is the purest, that is, has most oxygen in it, the more we breathe of that out-door air, the more nourishment do we require; and the more nourishment a man requires, the better appetite he has: hence, to get a *natural* appetite, a man must go out of doors; and as it is very tiresome to be out of doors, unless one is doing something, and, as if we do something, it had better be of some account, therefore, whoever wants to whet up his appetite, had better spend his time out of doors, doing something useful. A very *perspicacious ratiocination!*

All this seems very rational and very right. Then why do we not act up to it? Why pursue the very opposite course, and instead of going out of doors when we feel dull, and stupid, and cross, and desponding, loll about the house, as blue as indigo, with not a word or smile for anybody? Having no appetite, we bethink ourselves of "tonics." The reckless take wine, or brandy, or vulgar beer; the conscientious do worse, and take physic, calling it "bitters," tansy, dogwood, quinine, and such "*simple things*," 'specially the quinine, which has helped to invalid and kill more people than would make a monument sky-high.

Well, what is the result of these "tonics?" They make us feel better—for a while—give us an appetite for more than we can digest, and being imperfectly digested, the blood which it makes is not only imperfect as to quality, it is too great in quantity; but it is in the body, and must crowd itself somewhere, *always* selecting the weaker part, which, in most cases, is the head!—very natural that—and there is headache, dullness—never was much brightness in that head anyhow—in fact, it amounts to stupidity, and such persons being naturally stupid, and making themselves artificially so, they have a double right to the title: as the youth had to a diploma, who graduated at two colleges, and became as the calf did which sucked two cows—a very great calf!

Therefore, never eat by rule. Never eat at one meal as much as you did at the corresponding one the day before, simply because that was your usual quantity; but eat according to your appetite. If you have no appetite, eat nothing until you do. If you are in a hurry for that appetite, and time is valuable to you, do not attempt to whet it up by stimulating food, by exciting drinks, or forcing tonics, but bring it about in a natural way, by moderate and continuous exercise in the open air, in something that is interesting, exciting, and in itself useful. Violent spasmodic exercise is injurious, and even dangerous to sedentary persons. Hence, we are opposed to *gymnasiums*, unless superintended by intelligent men, practical physiologists. Let it be remembered, as a truth which cannot be denied, that a given amount of violent exercise taken within an hour will do many times the good, if scattered continuously over a space of five hours, without any of the danger that pertains to the former, especially as to feeble persons. All exercise carried to severe fatigue, is an injury; better have taken none.

